# Clinical decision support systems to optimize adherence to anticoagulant guidelines in patients with atrial fibrillation: a systematic review and meta-analysis of randomized controlled trials

**DOI:** 10.1186/s12959-024-00614-7

**Published:** 2024-05-28

**Authors:** Ahmed Mazen Amin, Ramy Ghaly, Mohamed T. Abuelazm, Ahmed A. Ibrahim, Mohammad Tanashat, Moumen Arnaout, Obieda Altobaishat, Ahmed Elshahat, Basel Abdelazeem, Sudarshan Balla

**Affiliations:** 1 Faculty of Medicine, Mansoura University, Mansoura, Egypt; 2 Internal Medicine, University of Missouri-Kansas City, Kansas City, MO USA; 3 Faculty of Medicine, Tanta University, Tanta, Egypt; 4 Faculty of Medicine, Menoufia University, Menoufia, Egypt; 5 Faculty of Medicine, Yarmouk University, Irbid, Jordan; 6 Faculty of Medicine, Aleppo University, Aleppo, Syria; 7 Faculty of Medicine, Jordan University of Science and Technology, Irbid, Jordan; 8 Faculty of Medicine, Al-Azhar University, Cairo, Egypt; 9 Department of Cardiology, West Virginia University, Morgantown, WV USA

**Keywords:** Atrial fibrillation, Oral anticoagulation, Electronic notifications, Electronic alerts.

## Abstract

**Background:**

Clinical decision support systems (CDSS) have been utilized as a low-cost intervention to improve healthcare process measures. Thus, we aim to estimate CDSS efficacy to optimize adherence to oral anticoagulant guidelines in eligible patients with atrial fibrillation (AF).

**Methods:**

A systematic review and meta-analysis of randomized controlled trials (RCTs) retrieved from PubMed, WOS, SCOPUS, EMBASE, and CENTRAL through August 2023. We used RevMan V. 5.4 to pool dichotomous data using risk ratio (RR) with a 95% confidence interval (CI). PROSPERO ID: CRD42023471806.

**Results:**

We included nine RCTs with a total of 25,573 patients. There was no significant difference, with the use of CDSS compared to routine care, in the number of patients prescribed anticoagulants (RR: 1.06, 95% CI [0.98, 1.14], *P* = 0.16), the number of patients prescribed antiplatelets (RR: 1.01 with 95% CI [0.97, 1.06], *P* = 0.59), all-cause mortality (RR: 1.19, 95% CI [0.31, 4.50], *P* = 0.80), major bleeding (RR: 0.84, 95% CI [0.21, 3.45], *P* = 0.81), and clinically relevant non-major bleeding (RR: 1.05, 95% CI [0.52, 2.16], *P* = 0.88). However, CDSS was significantly associated with reduced incidence of myocardial infarction (RR: 0.18, 95% CI [0.06, 0.54], *P* = 0.002) and cerebral or systemic embolic event (RR: 0.11, 95% CI [0.01, 0.83], *P* = 0.03).

**Conclusion:**

We report no significant difference with the use of CDSS compared to routine care in anticoagulant or antiplatelet prescription in eligible patients with AF. CDSS was associated with a reduced incidence of myocardial infarction and cerebral or systemic embolic events.

**Supplementary Information:**

The online version contains supplementary material available at 10.1186/s12959-024-00614-7.

## Introduction

Atrial fibrillation (AF) is the most prevalent arrhythmia worldwide [1–3]. AF increases the risk for stroke up to fivefold, contributing to up to 25% of all strokes [4, 5]. Societal guidelines in the U.S recommend using the CHA_2_DS_2_-VASc score to quantify the annual stroke risk and guide oral anticoagulation therapy (OAC) with either direct oral anticoagulants (DOACs) or vitamin K antagonists (VKA). A CHA_2_DS_2_-VASc score of one in men and two in women warrants prescribing OAC to reduce the risk of thromboembolic events. However, the CHA_2_DS_2_-VASc score is not recommended in AF patients with moderate to severe mitral stenosis or mechanical heart valves, where VKA is warranted [1, 6].

In a meta-analysis including 28,044 patients, prescribing VKA resulted in a 64% relative risk reduction (RRR) of stroke in patients with AF [7]. DOACs, including dabigatran, rivaroxaban, and apixaban, showed at least similar stroke prevention efficacy with a favorable safety profile [8–10]. Despite the significant RRR of stroke by OAC, there has been underutilization of OAC in AF patients [11–16]. In an observational study involving 94,474 patients who had experienced an acute ischemic stroke and had a history of AF, it was found that 84% of them had not been prescribed OAC before the occurrence of the stroke [17].

Clinical decision support systems (CDSS) have been increasingly utilized as a low-cost intervention to improve healthcare process measures; however, their impact on improving clinical outcomes remains controversial [18]. A randomized clinical trial (RCT) showed that an alert system increased the prescription of deep vein thrombosis (DVT) prophylaxis and reduced thromboembolism rates by 41% among hospitalized patients [19]. On the other hand, an alert system did not improve clinical outcomes in hospitalized patients with acute kidney injury [20].

Several RCTs were conducted to study the utility of CDSS and alert systems to improve OAC prescription among AF patients to reduce the risk of stroke and systemic embolism potentially.

We conducted this systematic review and meta-analysis of RCTs to investigate the efficacy of CDSS versus routine care regarding adherence to OAC prescription guidelines and stroke prevention in patients with AF.

## Methodology

### Protocol Registration

The study’s protocol was registered in PROSPERO with the identification number CRD42023471806, following the Preferred Reporting Items for Systematic Review and Meta-analysis of Interventional Studies (PRISMA) statement [21] and the Cochrane Handbook for Systematic Reviews and Meta-Analysis [22] guidelines.

### Data sources & search strategy

PubMed, Web of Science, SCOPUS, EMBASE, and CENTRAL were searched by authors (A.M.A. and M.T.A.) through August 2023 without publication date, language, or geographical area restrictions. The search was done using [all field] with a mention of the usage of “alert” and “anticoagulant” in “Atrial Fibrillation” Patients. More details are in (Table S1).

### Eligibility criteria

Randomized controlled trials (RCTs) that met all of our PICO inclusion criteria were selected: population (P): AF patients; intervention (I): CDSS, including email alert, notification alert, and electronic alerts; comparison (C): patients treated with usual care or no intervention; outcomes (O): our primary outcome was OAC prescription, while our secondary outcomes were patients prescribed antiplatelets and patients prescribed VKA. Additionally, we assessed hard outcomes, including mortality, major bleeding, clinically relevant non-major bleeding, myocardial infarction, stroke/transient ischemic attack (TIA), and thromboembolic events. Exclusion criteria were as follows: primary studies other than RCTs, duplicate publications, reviews, and conference abstracts.

### Study selection

Four reviewers (M.T., A.E., O.A., and M.A.) initially screened the titles and abstracts independently using the Covidence platform. After erasing the duplicates, they independently screened the full texts in accordance with our previous eligibility criteria.

### Data extraction

Four reviewers (M.A., M.T., A.E., and O.A.) independently extracted data from the eligible studies. M.T.A. and A.M.A. resolved any conflicts. We used an Excel sheet: summary characteristics (study design, country, number of centers, blinding status, registry number, total participants, intervention details, control, participants were on OAC or not, primary outcome, and follow-up duration), baseline characteristics (number of patients in CDSS and control arms, age, gender (male), CHA_2_DS_2_VASc score, HAS-BLED score, and patients’ comorbidities (vascular disease, heart disease, diabetes mellitus, hypertension, stroke/transient ischemic attack (TIA), renal disease, liver disease, and prior bleeding). Additionally, the current study outcomes were the number of patients prescribed anticoagulant (OAC), patients prescribed antiplatelets, patients prescribed vitamin K antagonist (VKA), and proportions of why participants were not on OAC. In addition, hard clinical outcomes such as mortality, major bleeding, clinically relevant non-major bleeding, myocardial infarction, stroke/TIA, and thromboembolic events were assessed.

### Risk of Bias and Certainty of evidence

Four reviewers (M.A., M.T., A.E., and O.A.) independently used the Cochrane ROB2 tool [23] for quality assessment. The reviewers resolved any conflicts by consensus. We evaluated five domains, assessing the risk of bias due to randomization, deviation from CDSS, missing outcome data, measuring the outcome data, and selecting the reported results.

The Grading of Recommendations Assessment, Development, and Evaluation (GRADE) guidelines [24, 25] was used by M.T.A. to evaluate the certainty of evidence for each outcome.

### Statistical analysis

RevMan v5.3 was used to run the statistical analysis [26]. To pool the results of dichotomous outcomes, we used the risk ratio (RR), while for the continuous outcomes, we used the mean difference (MD), both with a 95% confidence interval (CI). We performed both the Chi-square and I-square tests to evaluate heterogeneity, where the Chi-square test detects the presence of heterogeneity, and the I-square test evaluates its degree. I-square was interpreted In accordance with the Cochrane Handbook (chapter nine) [22]. as follows: heterogeneity is not significant for 0–40%, moderate for 30–60%, substantial for 50–90%, and considerable for 75–100%. We considered an alpha level below 0.1 for the Chi-square test to detect significant heterogeneity. A leave-one-out sensitivity analysis was employed to resolve the heterogeneity by excluding each study one time from the pooled analyzed studies.

Rstudio (version 4.2.2) was used to conduct a meta-analysis of prevalence using the random effect model with a 95% confidence interval. The I-square test was used to assess for heterogeneity, with I^2^ > 50% considered to be of significant heterogeneity.

## Results

### Search results and study selection

**Fig. 1:**
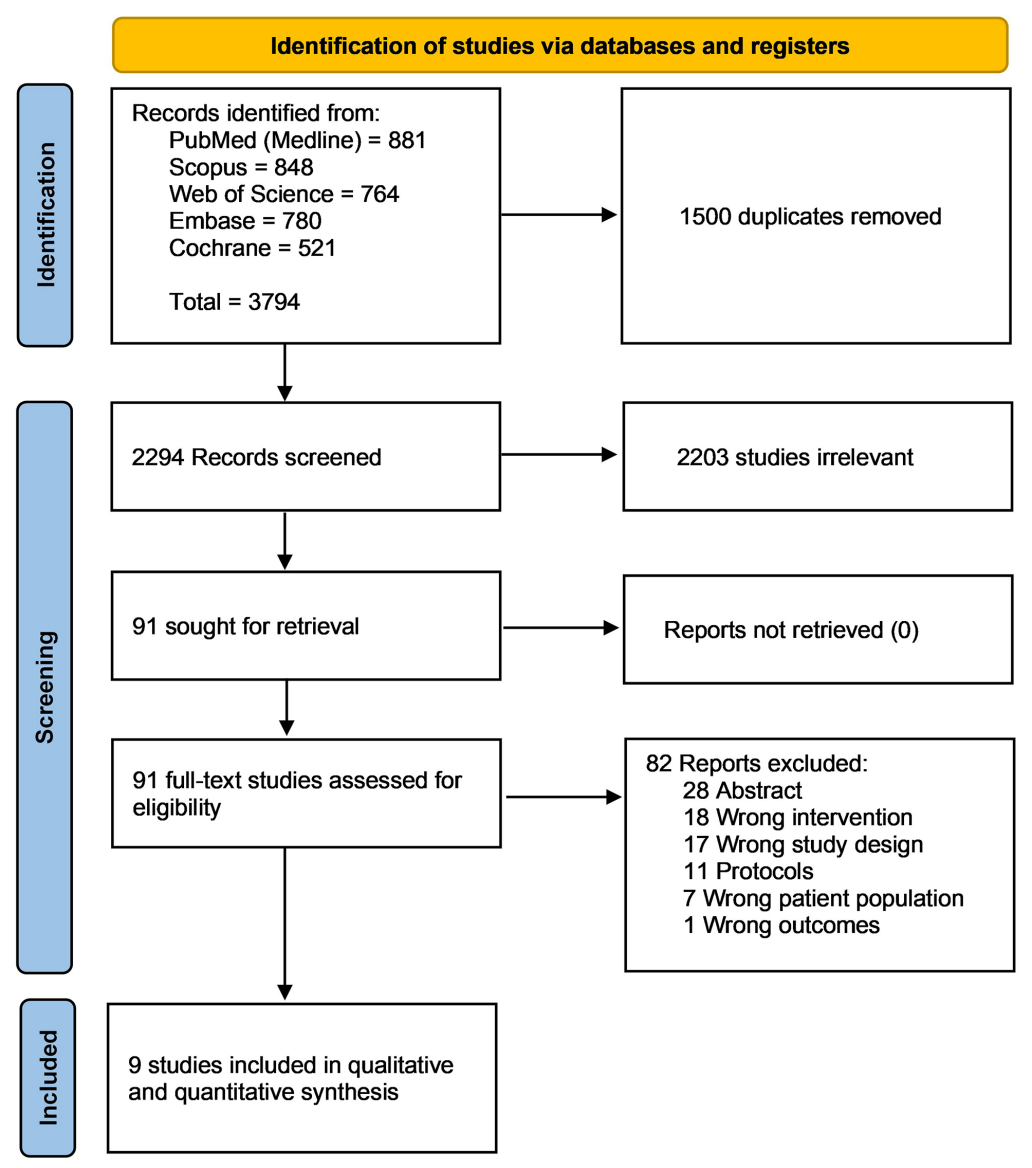
PRISMA flow chart of the screening process

Our literature search retrieved 3,794 unique records. One thousand-five hundred records were removed as duplicates. After title and abstract screening, 91 studies were eligible for full-text screening. Finally, nine studies were included in this systematic review and meta-analysis. The PRISMA flowchart for study selection is shown in (Fig. 1). We have excluded Guo et al. trial [27] due to differences in the intervention compared to our included RCTs’ intervention. Patients could upload reports and pictures of the events, unlike our interventions, which are Electronic Medical Record (EMR) based CDSS.

### Characteristics of included studies

Nine randomized controlled trials [28–36] were included in the meta-analysis with 25,573 AF patients. All the included studies accessed our primary outcome, the number of patients on OAC. The follow-up duration in those studies ranged from three months to 12 months. These studies were conducted in five countries, mainly in the USA (five trials). The summary and baseline characteristics of the included studies are shown in (Tables 1 and 2). More details about the baseline trials’ participants’ comorbidities and CDSS characteristics are outlined in (Tables S2 and S3).

**Table 1:** Summary characteristics of the included RCTs.

Study ID	Study Design	Country	Total Participants	Intervention	Control	Already on OAC	Primary Outcome	Follow-up duration	
**Arts et al. 2017** [28]	Single center, RCT	Netherlands	781	A real-time CDSS for a single EHR system	Received no messages	BOTH	The effect of the intervention on the proportion of patients with AF treated in accordance with the guideline between the intervention and control groups.	Nine months	
**Ashburner et al. 2018** [29]	Single center, RCT	USA	2336	A physician notification alert and survey	Usual care	NO	the proportion of patients prescribed oral anticoagulants at three months in the intervention group in comparison with the control group	Three months	
**Bajorek et al. 2016** [30]	Multi-center, RCT	Australia	393	computerized antithrombotic risk assessment tool	Usual care	BOTH	Change in anticoagulants and antiplatelets description	12 months	
**Chaturvedi et al. 2019** [31]	Multi-center, RCT	USA	309	electronic alert (EA) embedded in the electronic health record	Usual care	NO	comparing OAC consumption in active intervention locations to usual care settings	Six months	
**Kapoor et al. 2020 (SUPPORT-AF II)** [32]	Single-center, RCT	USA	5475	electronic profiling/messaging combined with academic detailing	No intervention	BOTH	Feasibility (how often providers in the intervention group read the emails) and effectiveness (change in anticoagulation status)	Seven months	
**Karlsson et al. 2018 (CDS-AF)** [33]	Multi-center, RCT	Sweden	14,134	CDS &alert for physicians	Usual care	BOTH	proportion of patients eligible for stroke prophylaxis who were prescribed anticoagulant therapy 12 months after study initiation.	12 months	
**Piazza et al. 2019 (AF-ALERT)** [34]	RCT	USA	458	Alert-base CDS	No notification	NO	frequency of anticoagulant prescription	Three months	
**Piazza et al. 2023 (AF-ALERT2)** [35]	RCT	USA	798	Alert-based CDS	No notification	NO	frequency of anticoagulant prescription	Three months	
**Silbernagel et al. 2016** [36]	RCT	Switzerland	889	computer-based electronic alert system	no alert (usual care)	NO	rate of adequate OAC prescription at hospital discharge	N/A	

RCT: randomized controlled trial; AF: atrial fibrillation; CDSS: clinical decision support system; OAC: oral anticoagulant; N/A.: not available

**Table 2:** Baseline characteristics of the participants

Study ID	Number of patients in each group		Age (Years), Mean (SD)		Gender (Male), *N*. (%)		CHA2DS2VASC, Mean (SD)		HAS-BLED score, Mean (SD)	
	Intervention	Control	Intervention	Control	Intervention	Control	Intervention	Control	Intervention	Control
**Arts et al. 2017** [28]	522	259	72.13 (12.46)	74.61 (13.63)	N/A	N/A	3 (1.72)	3.06 (1.8)	N/A	N/A
**Ashburner et al. 2018** [29]	972	1364	75.7 (11.1)	76.3 (11.5)	490(50.4)	725(53.1)	4.2 (1.7)	4.2 (1.6)	N/A	N/A
**Bajorek et al. 2016** [30]	206	187	78.2 (7.1)	77.7 (7)	113(54.9)	101(54)	N/A	N/A	N/A	N/A
**Chaturvedi et al. 2019** [31]	164	145	69.85 (12.53)	70.57 (11.89)	93(56.7)	81(55.9)	3.78 (1.87)	3.1 (1.59)	N/A	N/A
**Kapoor et al. 2020 (SUPPORT-AF II)** [32]	3578	1897	N/A	N/A	1940(54.2)	1077(56.8)	N/A	N/A	N/A	N/A
**Karlsson et al. 2018 (CDS-AF)** [33]	7764	6370	N/A	N/A	4042(54.4)	3269(54)	4(1.48288)	4(1.4892)	N/A	N/A
**Piazza et al. 2019 (AF-ALERT)** [34]	248	210	73.5(11.8)	73.3(13)	136(54.8)	117(55.7)	4(1.33)	4(1.166)	3(1.166)	3(1.1667)
**Piazza et al. 2023 (AF-ALERT2)** [35]	395	403	73.7(11.7)	72(11.9)	225(57)	242(60.1)	3.66(2.23)	3.66(2.23)	3.66(2.23)	3(1.48)
**Silbernagel et al. 2016** [36]	455	434	74.4(10.9)	73.3(11.8)	300(65.9)	292(67.3)	N/A	N/A	N/A	N/A

N., number; SD, standard deviation; N/A: not available

### Risk of Bias and Certainty of evidence

**Fig. 2:**
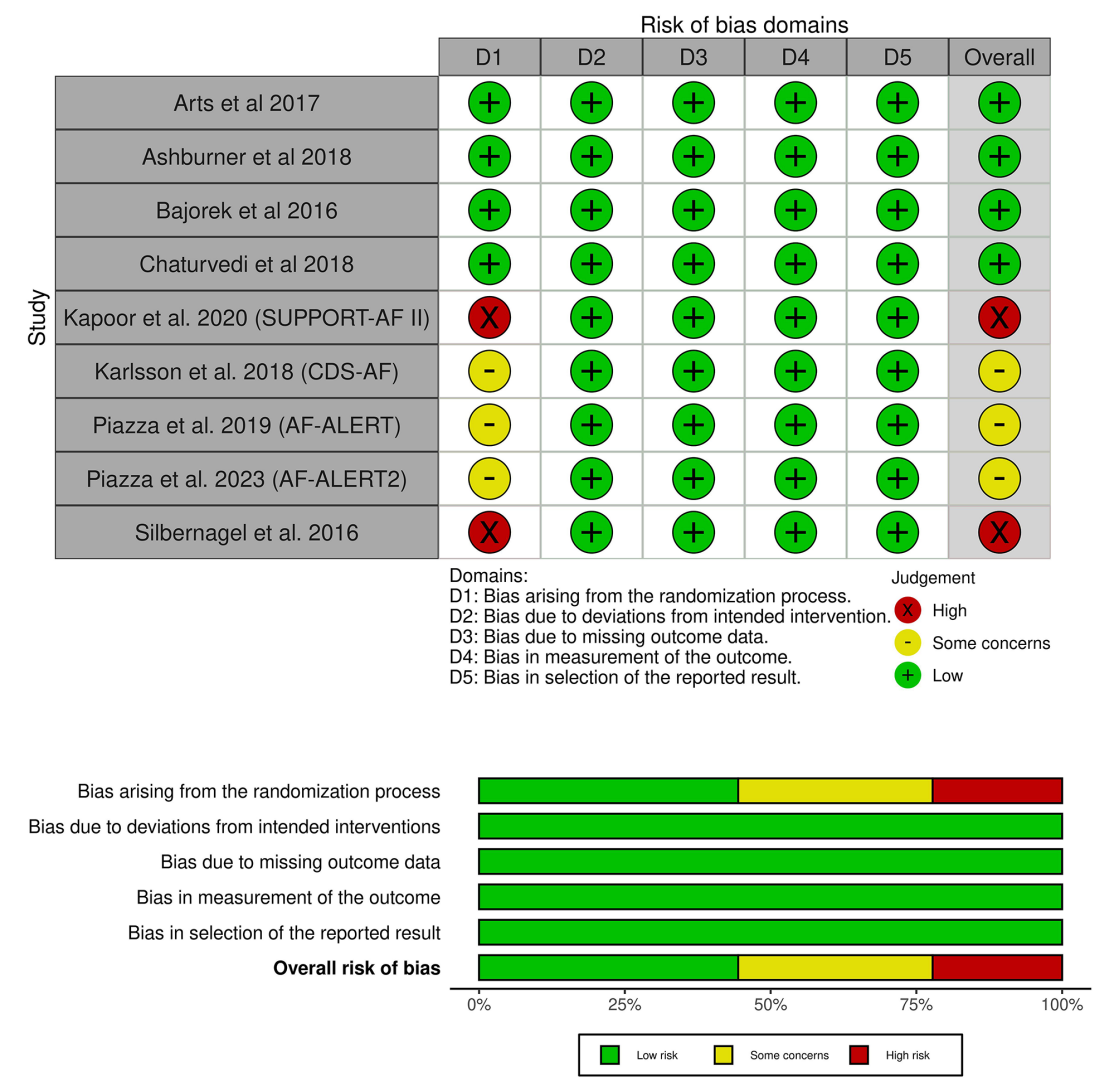
Quality assessment of risk of bias in the included trials. The upper panel presents a schematic representation of risks (low = green, unclear = yellow, and high = red) for specific types of biases of each study in the review. The lower panel presents risks (low = green, unclear = yellow, and high = red) for the subtypes of biases of the combination of studies included in this review

We assessed the quality of included studies according to the Cochrane risk of bias 2 tool, as shown in (Fig. 2). Four included trials had a low risk of randomization process bias (Arts et al. 2017, Ashbumer et al. 2018, Bajorek et al. 2016, and Chaturvedi et al. 2018), three had some concerns (Karlsson et al. 2018, Piazza et al. 2019 and Piazza et al. 2023), and two had a high risk (Kapoor et al. 2020 and Silbemagel et al. 2016). All the included studies had a low risk of deviations from intended intervention bias, missing outcome data bias, measurement of the outcome bias, and selection of the reported result bias. Author judgments are further clarified in (Table S4). Certainty of evidence is demonstrated in a GRADE evidence profile (Table 3).

**Table 3:** GRADE evidence profile

Certainty assessment							Summary of findings				
Participants (studies) Follow-up	Risk of bias	Inconsistency	Indirectness	Imprecision	Publication bias	Overall certainty of evidence	Study event rates (%)		Relative effect (95% CI)	Anticipated absolute effects	
							With Usual Care	With CDSS		Risk with Usual Care	Risk difference with CDSS
**number of patients on anticoagulant**											
24,567 (8 RCTs)	serious^a^	very serious^b^	not serious	not serious	none	⨁◯◯◯ Very low	6257/10,621 (58.9%)	9033/13,946 (64.8%)	**RR 1.04** (0.96 to 1.12)	589 per 1,000	**24 more per 1,000** (from 24 fewer to 71 more)
**number of patients on antiplatlets**											
5183 (6 RCTs)	serious^a^	not serious	not serious	not serious	none	⨁⨁⨁◯ Moderate	1651/2743 (60.2%)	1408/2440 (57.7%)	**RR 1.01** (0.97 to 1.06)	602 per 1,000	**6 more per 1,000** (from 18 fewer to 36 more)
**number of patients on vitamin k antagonist (aka.warfarin)**											
5027 (6 RCTs)	serious^a^	serious^c^	not serious	serious^d^	none	⨁◯◯◯ Very low	247/2679 (9.2%)	273/2348 (11.6%)	**RR 1.18** (0.84 to 1.66)	92 per 1,000	**17 more per 1,000** (from 15 fewer to 61 more)
**Adverse - All-cause mortality**											
1256 (2 RCTs)	serious^a^	serious^c^	not serious	very serious^d^	none	⨁◯◯◯ Very low	34/613 (5.5%)	33/643 (5.1%)	**RR 1.19** (0.31 to 4.50)	55 per 1,000	**11 more per 1,000** (from 38 fewer to 194 more)
**Adverse - Major bleed**											
1256 (2 RCTs)	serious^a^	serious^c^	not serious	very serious^d^	none	⨁◯◯◯ Very low	11/613 (1.8%)	9/643 (1.4%)	**RR 0.84** (0.21 to 3.45)	18 per 1,000	**3 fewer per 1,000** (from 14 fewer to 44 more)
**Adverse - Clinically relevant non-major bleed**											
1256 (2 RCTs)	serious^a^	not serious	not serious	very serious^d^	none	⨁◯◯◯ Very low	14/613 (2.3%)	16/643 (2.5%)	**RR 1.05** (0.52 to 2.16)	23 per 1,000	**1 more per 1,000** (from 11 fewer to 26 more)
**Adverse - Myocardial infarction**											
1256 (2 RCTs)	serious^a^	not serious	not serious	very serious^d^	none	⨁◯◯◯ Very low	20/613 (3.3%)	4/643 (0.6%)	**RR 0.18** (0.06 to 0.54)	33 per 1,000	**27 fewer per 1,000** (from 31 fewer to 15 fewer)
**Adverse - Stroke/TIA or systemic embolic event**											
16,056 (3 RCTs)	serious^a^	not serious	not serious	very serious^d^	none	⨁◯◯◯ Very low	8/7121 (0.1%)	0/8935 (0.0%)	**RR 0.11** (0.01 to 0.83)	1 per 1,000	**1 fewer per 1,000** (from 1 fewer to 0 fewer)

CI: confidence interval; RR: risk ratio

Explanations

a. Karlsson et al. 2018, Piazza et al. 2019 and Piazza et al. 2023 had some concerns of selection biaas, while Kapoor et al. 2020 and Silbemagel et al. 2016 had a high risk of selection bias

b. I-square test > 75%

c. I-square test > 50%

d. Wide confidence interval that does not exclude the risk of appreciable benefit/harm

### Primary outcome: number of patients on OAC

**Fig. 3:**
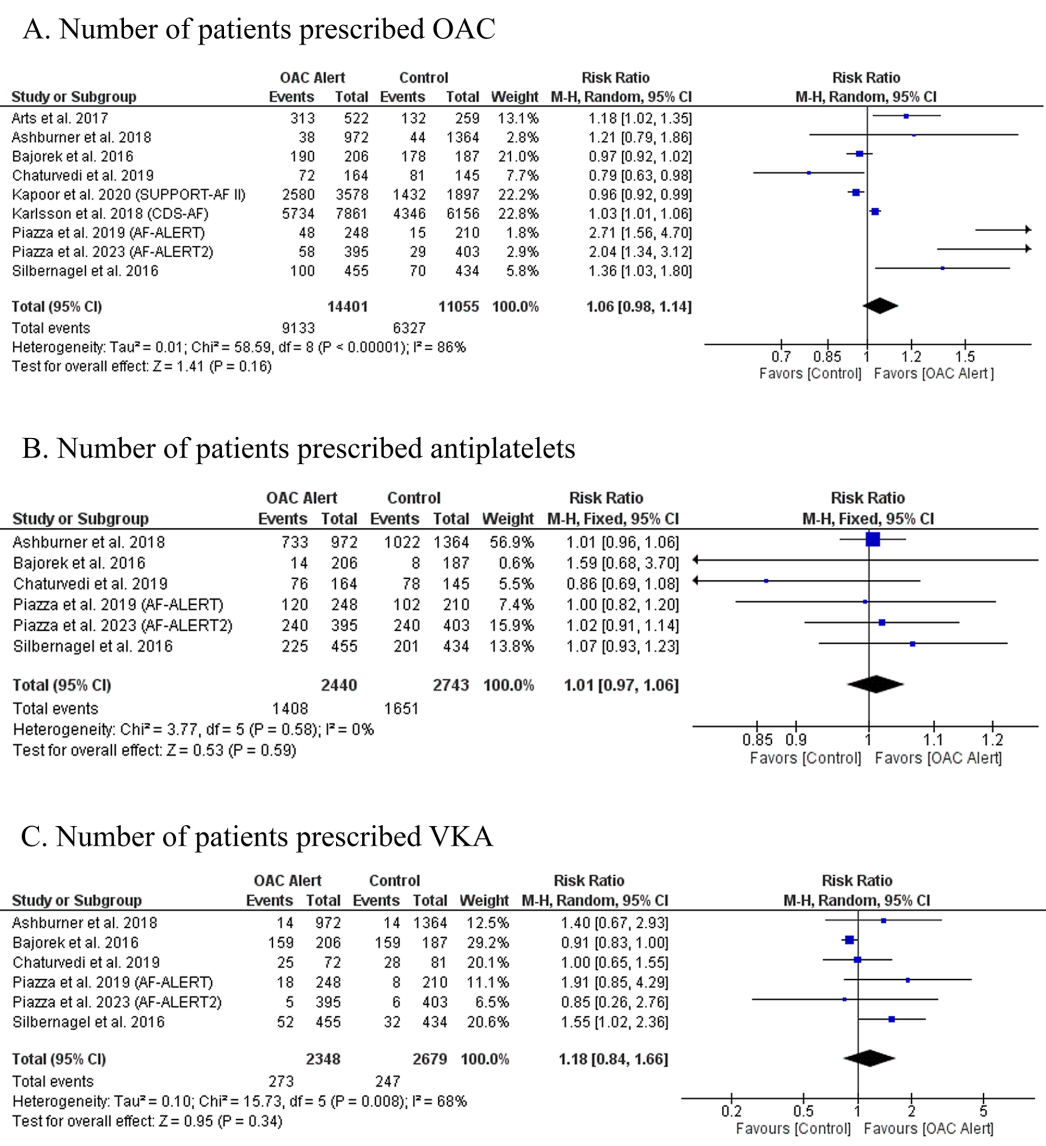
Forest plot of the primary outcome (prescription of OAC) with the secondary outcome (prescription of antiplatelet and VKA), RR: risk ratio, CI: confidence interval

There was no significant difference in the number of patients prescribed OAC between CDSS compared to routine care (RR: 1.06 with 95% CI [0.98, 1.14], *P* = 0.16) (Fig. 3-A). The pooled studies were heterogeneous (I^2^ = 87%, *P* < 0.00001). Heterogeneity was not resolved by leave-one-out sensitivity analysis (Table S5).

### Secondary outcomes

#### Efficacy outcomes

There was no significant difference whether using CDSS or not in the number of patients prescribed antiplatelets (RR: 1.01 with 95% CI [0.97, 1.06], *P* = 0.59) (Fig. 3-B) and the number of patients prescribed VKA (RR: 1.18 with 95% CI [0.84, 1.66], *P* = 0.34) (Fig. 3-C).

The pooled studies were homogenous in number of patients prescribed antiplatelets (I^2^ = 0%, *P* = 0.58). However, pooled studies were heterogeneous in number of patients prescribed VKA (I^2^ = 68%, *P* = 0.008). Regarding the number of patients prescribed VKA, heterogeneity was best resolved by excluding Bajorek et al. 2016 and Silbernagel et al. 2016 (I^2^ = 0%, *P* = 0.48), (I^2^ = 36%, *P* = 0.18), respectively (Table S5).

#### Reasons why participants were not on OAC

**Fig. 4:**
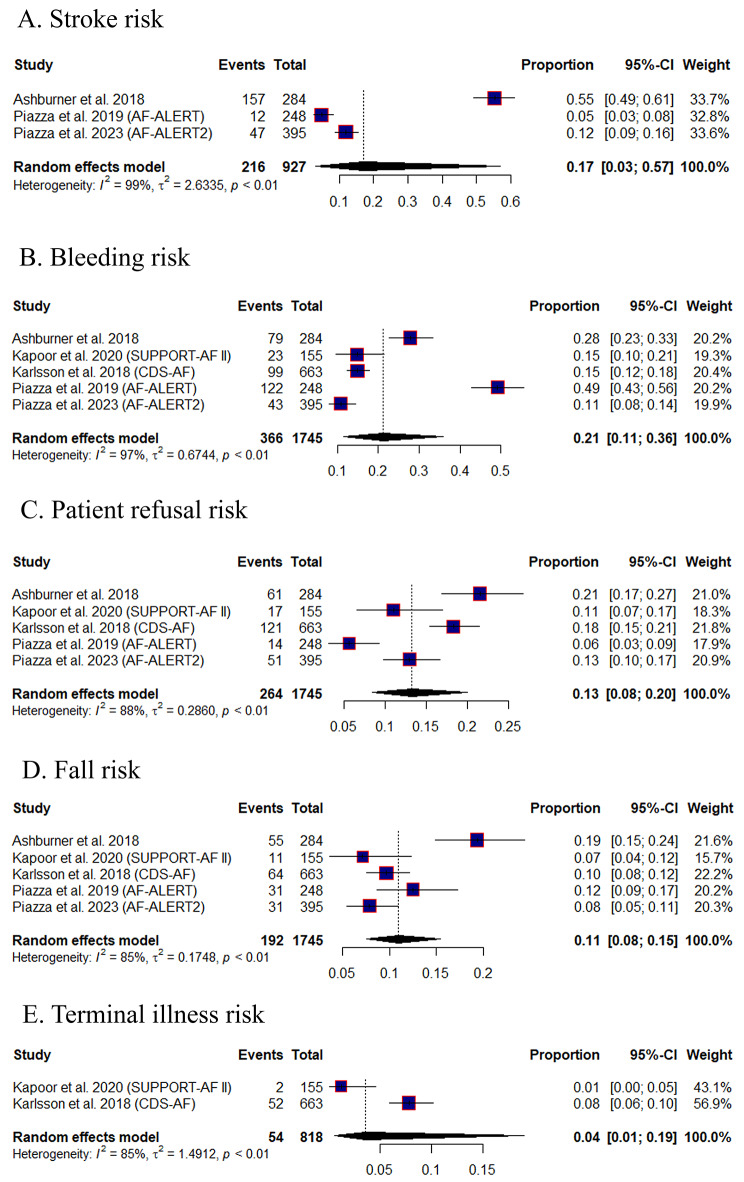
Forest plots of the meta proportion of why participants were not on OAC, CI: confidence interval

The pooled prevalence of stroke risk, from three studies (*n* = 927), was 17% (95% CI [0.03, 0.57], I^2^ = 99%) (Fig. 4-A), bleeding risk, from five studies (*n* = 1745), was 21% (95% CI [0.11, 0.36], I^2^ = 97%) (Fig. 4-B), patient refusal, from five studies (*n* = 1745), was 13% (95% CI [0.08, 0.20], I^2^ = 88%) (Fig. 4-C), fall risk, from five studies (*n* = 1745), was 11% (95% CI [0.08, 0.15], I^2^ = 85%) (Fig. 4-D), and terminal illness or hospice, from two studies (*n* = 818), was 4% (95% CI [0.01, 0.19], I^2^ = 85%) (Fig. 4-E).

#### Hard clinical outcomes

**Fig. 5:**
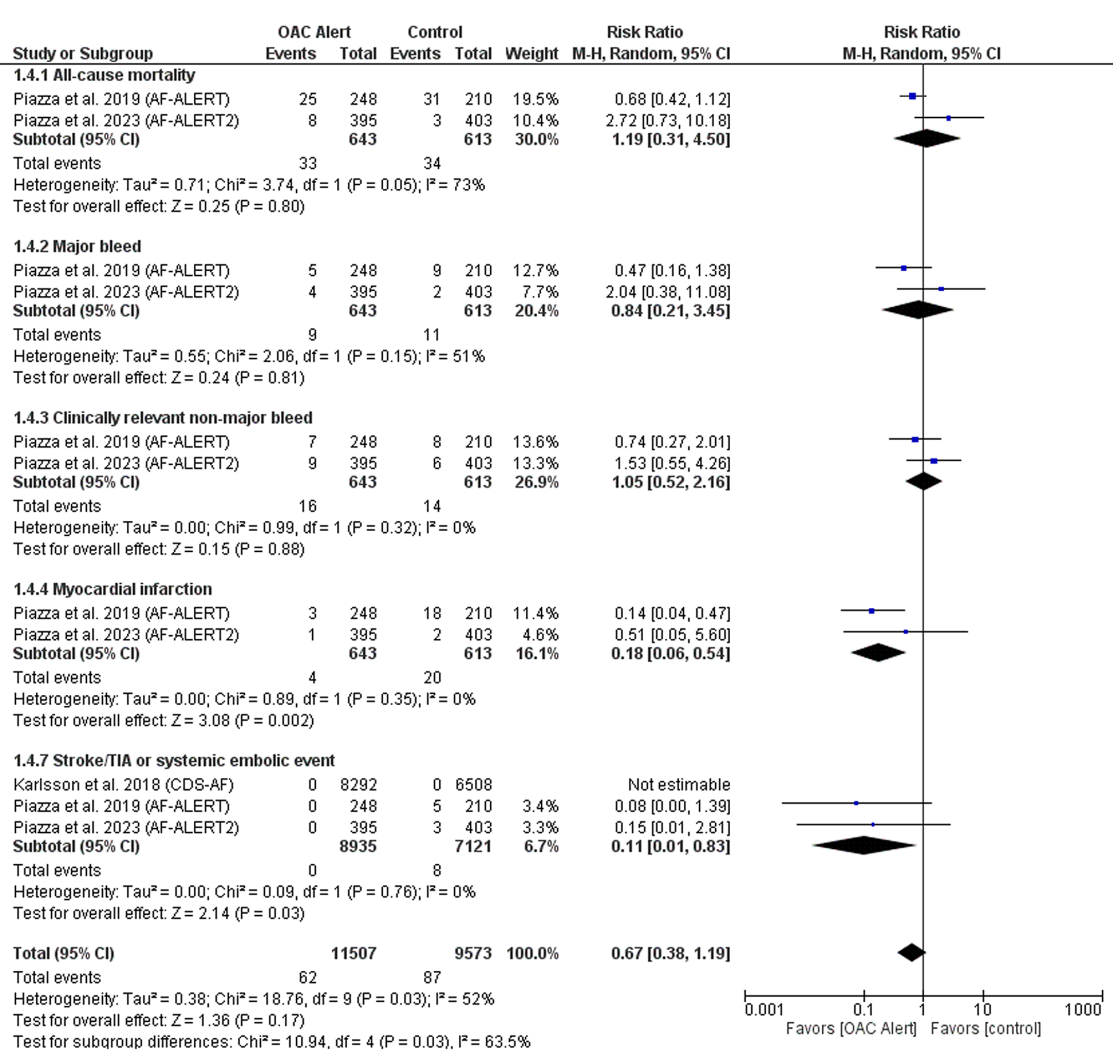
Forest plot of the clinical hard outcomes, RR: risk ratio, CI: confidence interval

CDSS was significantly associated with a reduced incidence of myocardial infarction (RR: 0.18 with 95% CI [0.06, 0.54], *P* = 0.002) and reduced incidence of stroke/TIA or systemic embolic event (RR: 0.11 with 95% CI [0.01, 0.83], *P* = 0.03). However, there was no significant difference between CDSS compared to routine care in the incidence of all-cause mortality (RR: 1.19 with 95% CI [0.31, 4.50], *P* = 0.80), the incidence of major bleeding (RR: 0.84 with 95% CI [0.21, 3.45], *P* = 0.81), and the incidence of clinically relevant non-major bleeding (RR: 1.05 with 95% CI [0.52, 2.16], *P* = 0.88) (Fig. 5).

The pooled studies were homogenous in clinically non-relevant major bleed (I^2^ = 0%, *P* = 0.32), myocardial infarction (I^2^ = 0%, *P* = 0.35), and stroke/TIA or thromboembolic event (I^2^ = 0%, *P* = 0.76). However, pooled studies were heterogeneous for all-cause mortality (I^2^ = 73%, *P* = 0.05) and major bleeding (I^2^ = 51%, *P* = 0.15).

## Discussion

In this systematic review and meta-analysis of nine RCTs involving 25,573 AF patients, we investigated the efficacy of CDSS in oral anticoagulant prescriptions for eligible patients with AF. Key findings include: (1) CDSS was not associated with a significant difference in OAC and antiplatelet prescription rates between CDSS and routine care. (2) CDSS use was associated with significantly reduced rates of myocardial infarction and cerebral or systemic embolic events (3) There was no significant difference in all-cause mortality, major bleeding, and clinically relevant non-major bleeding between CDSS use and routine care.

The Atrial Fibrillation Better Care (ABC) pathway was developed for integrated care for AF patients. It includes a simple approach (avoid stroke, better symptom management, and cardiovascular and comorbidity risk reduction) that guides clinicians through decision-making. In the ABC pathway, prescribing an oral anticoagulant is only one piece of the integrated care approach [37]. The ABC pathway has been shown to improve outcomes in patients with AF [38, 39]. The above approach aligns with AF guidelines, which recommend a patient-centered, holistic approach, necessitating the involvement of multiple stakeholders in AF management decisions. Therefore, CDSS development and application contribute to a more holistic approach to caring for patients with AF, ensuring proper OACs management [40].

Multiple provider-directed interventions have been studied to improve anticoagulation rates among AF patients. For example, email notification to the provider was not associated with increased prescription rates [29]. In addition, the Support-AF trial found no benefit to email and inbox notifications [41]. Subsequently, electronic health record (EHR)-based CDSS alerts were developed to improve adherence to guidelines and increase anticoagulation rates in eligible AF patients.

Provider-directed EHR CDSS alerts were introduced as a cost-effective intervention to enhance work efficiency and clinical outcomes in inpatient and ambulatory settings. Kawamoto et al. described four essential features of CDSS, including “(a) provide decision support automatically as part of clinician workflow, (b) deliver decision support at the time and location of decision making, (c) provide actionable recommendations, and (d) use a computer to generate the decision support.” [42].

CDSS were studied in different clinical conditions with variable efficacy in improving clinical outcomes. Kucker et al. demonstrated increased use of DVT prophylaxis and reduced DVT and pulmonary embolism incidence with CDSS alerts (HR = 0.59, *P* = 0.001) [19]. Van Wyk et al. showed improved dyslipidemia screening and treatment with CDSS alerts [43]. On the other hand, Wilson et al. found no improvement in hospitalized patients with acute kidney injury [20]. Bright et al. conducted a large systematic review, including 148 trials assessing the efficacy of CDSS. Results demonstrated that process measures were often used as study endpoints rather than patient-related outcomes. 128/148 studies assessed healthcare process measures, while only 29/148 assessed clinical outcomes. There was a significant improvement in healthcare process measures, but evidence for clinical outcomes was sparse [18].

We report no significant difference in rates of anticoagulation prescription; however, this finding should be interpreted with caution due to significant heterogeneity among the included studies. An observational study by Osterland et al. reported no significant change in the trend of anticoagulant use before and after implementation of best practice advisory in eligible ambulatory AF patients [44]. Our results suggested a significant reduction in the incidence of myocardial infarction and cerebral or systemic emboli events. These results align with previous research on CDSS use across different diseases on improving clinical outcomes in other disease states, such as DVT and dyslipidemia [19, 43]. Additionally, there was no significant increase in bleeding complications. The efficacy and safety outcomes with the use of CDSS were variable. This is likely due to the limited duration of follow-up. The duration of follow-up of 3–12 months in the included studies may be too short to assess the impact on stroke or systemic embolism.

Barriers to CDSS tools include alert fatigue, increased number of clicks, time constraints, and clinician burnout [45, 46]. Arts et al. studied the physicians’ perspective of the CDSS; perceived barriers included workflow interruption, increased number of recommendations, and irrelevant recommendations [47]. Context-aware CDSS models could help address some of these barriers, possibly by limiting recommendations to a specific encounter [48].

In our study, reasons for not prescribing an OAC included bleeding risk (21%), patient refusal (13%), fall risk (11%), and terminal illness (4%). Incorporation of bleeding and thromboembolism risk scoring tools might be helpful to support clinical decision-making in high bleeding risk patients. Given the high rates of patient refusal, data from the IMPACT-AF trial suggests that patient-directed educational interventions could also lead to a significant increase in anticoagulation rates [49]. Patient refusal can be attributed to anticoagulation cost, repeated falls, concerns about bleeding, advanced age, and occupational implications [50].

### Limitations

Our review has the following limitations. Firstly, variations in baseline characteristics were noted among different study populations. Secondly, there was notable heterogeneity among studies in the effect size of various outcomes, including the number of patients on anticoagulants, all-cause mortality, and major bleeding. Thirdly, there was notable heterogeneity in CDSS interventions among different studies, which presents a valid concern when interpreting pooled meta-analysis results. The variation in CDSS interventions could explain some conflicting study results well. Fourthly, there was a considerable difference in study weights, which may significantly influence the contribution of certain studies to the pooled results. Given the aforementioned limitations, our study provides a systematic review to accurately interpret the results of individual studies. Moreover, true heterogeneity is expected in prevalence estimates due to differences in the time and place where the included studies were conducted. I^2^ statistics may not be discriminative and should be interpreted with caution in this case. In case of substantial heterogeneity, planned sensitivity analysis can help elucidate the factors associated with the variability among estimates [51]. Additionally, hard clinical outcomes were exclusively assessed by the same research group, Piazza et al., in AF-ALERT and AF-ALERT2, with the analysis involving a smaller patient cohort (*n* = 643). Moreover, challenges in CDSS implementation include a lack of medical informatics expertise in certain centers.

### Implications on Future Research

Future trials are required to investigate the impact of CDSS on clinical patient outcomes, particularly all-cause mortality and Major Adverse Cardiovascular Events (MACE). Additional research is warranted to define the optimal characteristics of CDSS, including the potential integration of artificial intelligence and machine learning to enhance its effectiveness. Future research should also explore the physician perspective, with attention to potential issues such as alarm fatigue impacting CDSS usage and effectiveness in real-world settings.

## Conclusion

Our meta-analysis underscores CDSS’s potential to reduce the incidence of myocardial infarction and cerebral or systemic embolic events in patients with AF. However, we report no significant difference in the rate of prescribing OAC and antiplatelets, all-cause mortality, major bleeding, or clinically relevant non-major bleeding. These insights can guide clinicians in optimizing CDSS use in AF management.

### Electronic supplementary material

Below is the link to the electronic supplementary material.

Supplementary Material 1

## Data Availability

No datasets were generated or analysed during the current study.
