# Supplementary material for: Clinical decision support systems to optimize adherence to anticoagulant guidelines in patients with atrial fibrillation: a systematic review and meta-analysis of randomized controlled trials
Source: Thromb J. 2024 May 28;22:45. doi: 10.1186/s12959-024-00614-7 (PMC11134712; doi:10.1186/s12959-024-00614-7)
Supplement: Supplementary file 1 — Supplementary Material 1 [file 12959_2024_614_MOESM1_ESM.docx]

**Supplementary material:**

**Title.**

**Clinical Decision Support Systems to Optimize Adherence to** **Anticoagulant Guidelines in Patients with Atrial Fibrillation: A Systematic Review and Meta-analysis of Randomized Controlled Trials.**

**Running Title.**

Clinical Decision Support Systems for Anticoagulant Guidelines.

**Authors.**

Ahmed Mazen Amin^1^, Ramy Ghaly^2^, Mohamed T. Abuelazm^3^, Ahmed A. Ibrahim^4^, Mohammad Tanashat^5^, Moumen Arnaout ^6^, Obieda Altobaishat^7^, Ahmed elshahat^8^, Basel Abdelazeem^9^, Sudarshan Balla^9^.

**Affiliations.**

1. Faculty of Medicine, Mansoura University, Mansoura, Egypt.
2. Internal Medicine, University of Missouri-Kansas City, Kansas City, MO, USA
3. Faculty of Medicine, Tanta University, Tanta, Egypt.
4. Faculty of Medicine, Menoufia University, Menoufia, Egypt.
5. Faculty of Medicine, Yarmouk University, Irbid, Jordan.
6. Faculty of Medicine, Aleppo University, Aleppo, Syria.
7. Faculty of Medicine, Jordan University of Science and Technology, Irbid, Jordan.
8. Faculty of Medicine, Al-Azhar University, Cairo, Egypt.
9. Department of Cardiology, West Virginia University, West Virginia, USA.

**Keywords.**

Atrial fibrillation; Oral anticoagulation; Electronic notifications; Electronic alerts.

**Contents:**

**Tables.**Table S1: Search strategy.

Table S2: Baseline characteristics (participants comorbidities).

Table S3: Description of risk of bias (ROB) assessment.

Table S4: Sensitivity analysis.

| Database | Search Terms | Search Field | Search Results |
| --- | --- | --- | --- |
| Pubmed | ((alert OR notification OR message OR mail* OR Computerized OR computerised OR "order entry" OR CPOE OR reminder* OR "decision support*" OR "decision aid*" OR "decision tool*" OR "Electronic prescribing" OR CDS*) AND ("atrial fibrillation" OR AF OR AFib) AND (anticoagula* OR "direct oral anticoagulant*" OR DOAC* OR NOAC* OR apixaban OR rivaroxaban OR edoxaban OR "Direct thrombin inhibitor*" OR Dabigatran OR "anti-vitamin K" OR warfarin OR "Vitamin K antagonist" OR VKA)) | All Fields | 881 |
| Cochrane | ((alert OR notification OR mail* OR Computerized OR computerised OR "order entry" OR CPOE OR reminder* OR (decision NEXT support*) OR (decision NEXT aid*) OR (decision NEXT tool*) OR "Electronic prescribing" OR CDS) AND ("atrial fibrillation" OR AF OR AFib) AND (anticoagula* OR (direct NEXT oral NEXT anticoagulant*) OR DOAC* OR NOAC* OR apixaban OR rivaroxaban OR edoxaban OR (Direct NEXT thrombin NEXT inhibitor*) OR Dabigatran OR "anti-vitamin K" OR warfarin OR "Vitamin K antagonist" OR VKA)) | All Fields | 521 |
| WOS | ((alert OR notification OR message OR mail* OR Computerized OR computerised OR "order entry" OR CPOE OR reminder* OR "decision support*" OR "decision aid*" OR "decision tool*" OR "Electronic prescribing" OR CDS*) AND ("atrial fibrillation" OR AF OR AFib) AND (anticoagula* OR "direct oral anticoagulant*" OR DOAC* OR NOAC* OR apixaban OR rivaroxaban OR edoxaban OR "Direct thrombin inhibitor*" OR Dabigatran OR "anti-vitamin K" OR warfarin OR "Vitamin K antagonist" OR VKA)) | All Fields | 764 |
| SCOPUS | TITLE-ABS-KEY ( ( ( alert OR notification OR message OR mail* OR computerized OR computerised OR "order entry" OR cpoe OR reminder* OR "decision support*" OR "decision aid*" OR "decision tool*" OR "Electronic prescribing" OR cds* ) AND ( "atrial fibrillation" OR af OR afib ) AND ( anticoagula* OR "direct oral anticoagulant*" OR doac* OR noac* OR apixaban OR rivaroxaban OR edoxaban OR "Direct thrombin inhibitor*" OR dabigatran OR "anti-vitamin K" OR warfarin OR "Vitamin K antagonist" OR vka ) ) ) | Title, Abstract, Keywords | 848 |
| EMBASE | #4.  #1 AND #2 AND #3                                           780  #3.  'atrial fibrillation':ti,ab,kw OR af:ti,ab,kw OR       200,289       afib:ti,ab,kw  #2.  anticoagula*:ti,ab,kw OR 'direct oral                  229,001       anticoagulant*':ti,ab,kw OR doac*:ti,ab,kw OR       noac*:ti,ab,kw OR apixaban:ti,ab,kw OR       rivaroxaban:ti,ab,kw OR edoxaban:ti,ab,kw OR       'direct thrombin inhibitor*':ti,ab,kw OR       dabigatran:ti,ab,kw OR 'anti-vitamin k':ti,ab,kw       OR warfarin:ti,ab,kw OR 'vitamin k       antagonist':ti,ab,kw OR vka:ti,ab,kw  #1.  alert:ti,ab,kw OR notification:ti,ab,kw OR             394,534       message:ti,ab,kw OR mail*:ti,ab,kw OR       computerized:ti,ab,kw OR computerised:ti,ab,kw OR       'order entry':ti,ab,kw OR cpoe:ti,ab,kw OR       reminder*:ti,ab,kw OR 'decision       support*':ti,ab,kw OR 'decision aid*':ti,ab,kw OR       'decision tool*':ti,ab,kw OR 'electronic       prescribing':ti,ab,kw OR cds*:ti,ab,kw  ....................................................... | All Fields | 780 |

**Table S1: Search Strategy.**

| **Study ID** | **Comorbidities N.(%)** | | | | | | | | | | | | | | | |
| --- | --- | --- | --- | --- | --- | --- | --- | --- | --- | --- | --- | --- | --- | --- | --- | --- |
|  | **Vascular disease** | | **Heart failure** | | **DM** | | **Hypertension** | | **Stroke/TIA** | | **Renal disease** | | **Liver disease** | | **Prior bleeding** | |
|  | **Intervention** | **control** | **Intervention** | **control** | **Intervention** | **control** | **Intervention** | **control** | **Intervention** | **control** | **Intervention** | **control** | **Intervention** | **control** | **Intervention** | **control** |
| **Arts et al. 2017** | N/A | N/A | N/A | N/A | N/A | N/A | N/A | N/A | N/A | N/A | N/A | N/A | N/A | N/A | N/A | N/A |
| **Ashburner et al. 2018** | 335(34.5) | 481(35.3) | 254(26.1) | 336(24.6) | 231(23.8) | 337(24.7) | 783(80.6) | 1084(79.5) | 307(31.6) | 410(30.1) | 99(10.2)) | 110(8.1) | 234(24.1) | 302(22.1) | N/A | N/A |
| **Bajorek et al. 2016** | N/A | N/A | 84 (40.8) | 16 (8.6) | 41 (19.9) | 37 (19.8) | 166 (80.6) | 102 (54.5) | 50 (24.3) | 22 (11.8) | N/A | N/A | N/A | N/A | N/A | N/A |
| **Chaturvedi et al. 2019** | 94 (57.3) | 53 (36.6) | 68 (41.5) | 21 (14.5) | 57 (34.8) | 39 (26.9) | 132 (80.5) | 101 (69.7) | 19(11.6) | 13(9) | N/A | N/A | N/A | N/A | 3 (1.8) | 2(1.4) |
| **Kapoor et al. 2020 (SUPPORT-AF II)** | 426(12) | 224(11.8) | 995(27.8) | 694(36.6) | 1108(31) | 574(30.3) | 3036(84.9) | 1591(83.9) | 415(11.6) | 236(12.4) | N/A | N/A | N/A | N/A | N/A | N/A |
| **Karlsson et al. 2018 (CDS-AF)** | 2630(34) | 2122(33) | 2735(35) | 2270(36) | 1763(23) | 1454(23) | 6082(78) | 4881(77) | 1435(18) | 1165(18) | 308(4) | 228(4) | N/A | N/A | N/A | N/A |
| **Piazza et al. 2019 (AF-ALERT)** | 98(39.5) | 61(29.1) | 103(41.5) | 93(44.3) | 76(30.7) | 77(36.7) | 214(86.3) | 185(88.1) | 56(22.6) | 34(16.2) | 26(10.5) | 29(13.8) | 7(2.8) | 5(2.4) | 83(33.5) | 87(41.4) |
| **Piazza et al. 2023 (AF-ALERT2)** | 172(43.5) | 148(36.7) | 114(28.9) | 137(34) | 86(21.8) | 99(24.6) | 336(85.1) | 366(90.8) | 49(12.4) | 57(14.1) | 19(4.8) | 21(5.2) | 10(2.5) | 7(1.7) | 118(29.9) | 123(30.5) |
| **Silbernagel et al. 2016** | 179(39.3) | 154(35.5) | 137(30.1) | 134(30.9) | 104(22.9) | 93(21.4) | 276(60.7) | 229(52.8) | N/A | N/A | 80(17.6) | 100(23) | 15(3.3) | 16(3.7) | 140(30.8) | 143(32.9) |

**Table S2: Baseline characteristics (participants comorbidities).**

N: number, TIA: transiet ischemic attack, DM: diabetes mellitus, N/A: not available.

| **Study** | **CDSS characteristics** |
| --- | --- |
| **Arts et al. 2017** | This study designed an EMR-based CDSS. The CDSS provided notifications containing CHA2DS2-VASc scores and recommendations, with two response buttons allowing providers to accept or decline the recommendation. |
| **Ashburner et al. 2018** | This study developed a physician notification alert and an accompanying survey distributed via email to physicians. The alert contained the CHA2DS2-VASc score and bleeding risk factors for individual patients. At the same time, the survey inquired about confirming AF diagnosis and anticoagulation status, soliciting input from decision-makers for anticoagulation, documenting reasons for not prescribing oral anticoagulants, and indicating the subsequent management steps post-alert reception. Additionally, the notification included a link to guidelines. |
| **Bajorek et al. 2016** | This study devised a web-based computerized antithrombotic risk assessment tool (CARAT) that evaluated a patient's stroke risk, bleeding risk, and medication safety concerns. Its purpose was to calculate the patient's estimated risk of stroke compared to bleeding, identify medication management issues, and provide therapy recommendations. |
| **Chaturvedi et al. 2018** | This study employed an EMR-based alert, which computed the CHA2DS2-VASc score of clinicians. |
| **Kapoor et al. 2020 (SUPPORT-AF II)** | This study devised an intervention combining electronic messaging with academic detailing. Electronic medical record-based messages were dispatched before appointments with AF patients eligible for anticoagulation but not on it, allowing providers to respond with explanations for their prescribing decisions. Additionally, optional academic detailing appointments were available. |
| **Karlsson et al. 2018 (CDS-AF)** | This study created an EMR-based CDSS alert for physicians managing AF patients with elevated thromboembolism risk (as per the CHA2DS2-VASc score) but not receiving anticoagulant therapy. |
| **Piazza et al. 2019 (AF-ALERT)** | This study devised an EMR CDSS alert that conveyed information on a patient's heightened stroke risk in AF, the absence of an active anticoagulation order, and the indication for anticoagulant therapy. Providers receiving the alert were presented with three options: access an order template for anticoagulation regimens, follow a link to evidence-based clinical practice guidelines, or proceed with order entry while explaining the omission of anticoagulation. |
| **Piazza et al. 2023 (AF-ALERT2)** | This study devised an EMR CDSS alert that conveyed information on a patient's heightened stroke risk in AF, the absence of an active anticoagulation order, and the indication for anticoagulant therapy. Providers receiving the alert were presented with three options: access an order template for anticoagulation regimens, follow a link to evidence-based clinical practice guidelines, or proceed with order entry while explaining the omission of anticoagulation. |
| **Silbernagel et al. 2016** | This study created an electronic alert system to identify hospitalized patients with AF who were not receiving anticoagulants. This system featured a CHA2DS2-VASc score calculation tool and recommendations for prescribing oral anticoagulants. |

**Table S3: Characteristics of the included CDSS.**

| **Study ID** | **Domain** | | **Decision** | | **Description** | |  |
| --- | --- | --- | --- | --- | --- | --- | --- |
|  |  |  |  |  |  |  |  |
| **Arts et al. 2017** | | Randomization proces | | Low risk | | Randomization was done at the GP practice level to reduce contamination bias. | |
|  |  | Deviations from intended interventions | | Low rsik | | participants are Specialists can't be blinded but appropate analysis was used. | |
|  |  | Missing outcome data | | Low risk | | Outcome data of nearly all randomized patients were available. | |
|  |  | Measurement of the outcome | | Low risk | | Appropriate tools were used to measure the outcome without difference between the two group arms. | |
|  |  | Selection of the reported result | | Low risk | | All outcomes, measurement tools, and analysis plans were pre-specified in the study protocol. | |
|  |  | **OVERALL** | | **LOW RISK** | | | |
| **Ashburner et al. 2018** | | Randomization process | | Low risk | | Patients of each physician were randomized using a computerized random number generator to the intervention of physician notification at baseline or to the usual care control group without physician notification (all physicians were also offered the option to receive notifications for control patients after the trial period). | |
|  |  | Deviations from intended interventions | | Low risk | | participants are Specialists can't be blinded but appropate analysis was used withoutt excluding any participant. | |
|  |  | Missing outcome data | | Low risk | | Outcome data were available for nearly all participants. | |
|  |  | Measurement of the outcome | | Low risk | | Appropriate tools were used to measure the outcome without difference between the two group arms. | |
|  |  | Selection of the reported result | | Low risk | | All outcomes, measurement tools, and analysis plans were pre-specified in the study protocol. | |
|  |  | **OVERALL** | | **LOW RISK** | | | |
| **Bajorek et al. 2016** | | Randomization process | | Low risk | | They were randomly allocated through computer-generated random allocation number sequences and then asked to recruit patients during a 3-month period, from January through March 2012. | |
|  |  | Deviations from intended interventions | | Low risk | | participants are Specialists can't be blinded but appropate analysis was used (intention-to-treat analysis) | |
|  |  | Missing outcome data | | Low risk | | Outcome data were available for nearly all participants. | |
|  |  | Measurement of the outcome | | Low risk | | Appropriate tools were used to measure the outcome without difference between the two group arms. | |
|  |  | Selection of the reported result | | Low risk | | All outcomes, measurement tools, and analysis plans were pre-specified in the study protocol. | |
|  |  | **OVERALL** | | **LOW RISK** | | | |
| **Chaturvedi et al. 2019** | | Randomization process | | Low risk | | The control site was determined using a random number generator without difference in baseline data between the two arms. | |
|  |  | Deviations from intended interventions | | Low risk | | participants are Specialists can't be blinded but appropate analysis was used. | |
|  |  | Missing outcome data | | Low risk | | Outcome data were available for nearly all participants. | |
|  |  | Measurement of the outcome | | Low risk | | Appropriate tools were used to measure the outcome without difference between the two group arms. | |
|  |  | Selection of the reported result | | Low risk | | data that produced this result analysed in accordance with a pre-specified analysis plan. | |
|  |  | **OVERALL** | | **LOW RISK** | | | |
| **Kapoor et al. 2020 (SUPPORT-AF II)** | | Randomization process | | High risk | | our study statistician generated the random allocation sequence using Statistical Analysis Software (SAS) in a 2.5 to 1 intervention: control allo cation, stratified by provider type (cardiology or PCP) with no information about concealment process and they stated,” We randomized more patients to intervention because we believed  that many providers would not end up meeting with us, and we wanted to ensure sufficient participation in the one-on one, academic detailing part of our intervention.” | |
|  |  | Deviations from intended interventions | | Low risk | | participants are Specialists can't be blinded but appropate analysis was used (intention-to-treat analysis) | |
|  |  | Missing outcome data | | Low risk | | Outcome data were available for nearly all participants, “overall helpfulness of initial session (96.7%), and overall helpfulness of follow-up session (83%).” | |
|  |  | Measurement of the outcome | | Low risk | | Appropriate tools were used to measure the outcome without difference between the two group arms. | |
|  |  | Selection of the reported result | | Low risk | | data that produced this result analysed in accordance with a pre-specified analysis plan. | |
|  |  | **OVERALL** | | **HIGH RISK** | | | |
| **Karlsson et al. 2018 (CDS-AF)** | | Randomization process | | Some concerns | | a core facility with statistical expertise at Linko¨ping University, Sweden, performed the randomization sequence. The clinics were stratified into 4 strata based on the number of patients listed at each clinic and current adherence to guidelines and there was no difference between baseline data between the two arms. | |
|  |  | Deviations from intended interventions | | Low risk | | participants are Specialists can't be blinded but appropate analysis was used (intention-to-treat analysis). | |
|  |  | Missing outcome data | | Low risk | | Outcome data were available for nearly all participants. | |
|  |  | Measurement of the outcome | | Low risk | | Appropriate tools were used to measure the outcome without difference between the two group arms. | |
|  |  | Selection of the reported result | | Low risk | | data that produced this result analysed in accordance with a pre-specified analysis plan. | |
|  |  | **OVERALL** | | **SOME CONCERNS** | | | |
| **Piazza et al. 2019 (AF-ALERT)** | | Randomization process | | Some concerns | | Eligible patients were randomly assigned to the alert and control groups by the CDS program, according to the provider identification number of the Attending Physician of record. Even and odd provider identification numbers were used to determine allocation to either the alert or non-alert (control) group. | |
|  |  | Deviations from intended interventions | | Low risk | | participants are Specialists can't be blinded but appropate analysis was used. | |
|  |  | Missing outcome data | | Low risk | | Outcome data were available for nearly all participants. | |
|  |  | Measurement of the outcome | | Low risk | | Appropriate tools were used to measure the outcome without difference between the two group arms. | |
|  |  | Selection of the reported result | | Low risk | | data that produced this result analysed in accordance with a pre-specified analysis plan. | |
|  |  | **OVERALL** | | **SOME CONCERNS** | | | |
| **Piazza et al. 2023 (AF-ALERT2)** | | Randomization process | | Some concerns | | The allocation was according to the provider identification number with no information about the concealment process. | |
|  |  | Deviations from intended interventions | | Low risk | | participants are Specialists can't be blinded but appropate analysis was used. | |
|  |  | Missing outcome data | | Low risk | | Outcome data were available for nearly all participants. | |
|  |  | Measurement of the outcome | | Low risk | | Appropriate tools were used to measure the outcome without difference between the two group arms. | |
|  |  | Selection of the reported result | | Low risk | | data that produced this result analysed in accordance with a pre-specified analysis plan. | |
|  |  | **OVERALL** | | **SOME CONCERNS** | | | |
| **Silbernagel et al. 2016** | | Randomization process | | High risk | | The Randomization was performed electronically by automatically generating a num ber between 1 and 65 535 for each eligible patient. Patients with odd numbers were randomized to the alert group, whereas patients with even numbers were randomized to the control group with no information about the concealment process. In addition, Both groups were balanced with respect to baseline characteristics with  the exception of a higher rate of systemic hypertension, a trend toward a higher rate of transient ischemic attack, and a trend toward a lower rate of renal dysfunction in the alert group. | |
|  |  | Deviations from intended interventions | | Low risk | | participants are Specialists can't be blinded but appropate analysis was used. | |
|  |  | Missing outcome data | | Low risk | | Outcome data were available for nearly all participants. | |
|  |  | Measurement of the outcome | | Low risk | | Appropriate tools were used to measure the outcome without difference between the two group arms. | |
|  |  | Selection of the reported result | | Low risk | | data that produced this result analysed in accordance with a pre-specified analysis plan. | |
|  |  | **OVERALL** | | **HIGH RISK** | | | |

**Table S4: Description of risk of bias (ROB) assessment.**

| Outcome | No. of  Participants (/) | No. of  trials | Quantitative data synthesis | | | | Heterogeneity analysis | | |
| --- | --- | --- | --- | --- | --- | --- | --- | --- | --- |
|  |  |  | MD | 95% CI | Z value | p-value | df | p-value | I2 (%) |
| **Number of patients on anticoagulant.** | | | | | | | | | |
| Omitting Arts et al. 2017 | 13879/10796 | 8 | 1.04 | [0.96, 1.12] | 0.90 | 0.37 | 8 | <0.001 | 87% |
| Omitting Ashburner et al. 2018 | 13429/9691 | 8 | 1.06 | [0.99, 1.14] | 1.29 | 0.20 | 8 | <0.001 | 88% |
| Omitting Bajorek et al. 2016 | 14195/10868 | 8 | 1.09 | [0.99, 1.20] | 1.84 | 0.07 | 8 | <0.001 | 87% |
| Omitting Chaturvedi et al. 2019 | 14195/10910 | 8 | 1.09 | [1.01, 1.16] | 1.95 | 0.05 | 8 | <0.001 | 87% |
| Omitting Kapoor et al. 2020 (SUPPORT-AF II) | 10823/9158 | 8 | 1.10 | [1.01, 1.20] | 1.98 | 0.05 | 8 | <0.001 | 84% |
| Omitting Karlsson et al. 2018 (CDS-AF) | 6540/4899 | 8 | 1.11 | [0.99, 1.23] | 1.72 | 0.09 | 8 | <0.001 | 88% |
| Omitting Piazza et al. 2019 (AF-ALERT) | 14153/10845 | 8 | 1.05 | [0.98, 1.12] | 0.89 | 0.37 | 8 | <0.001 | 85% |
| Omitting Piazza et al. 2023 (AF-ALERT2) | 14006/10652 | 8 | 1.04 | [0.98, 1.12] | 0.84 | 0.40 | 8 | <0.001 | 86% |
| Omitting Silbernagel et al. 2016 | 13946/10621 | 8 | 1.05 | [0.98, 1.13] | 0.95 | 0.34 | 8 | <0.001 | 87% |
| **Number of patients on vitamin k antagonist (warfarin).** | | | | | | | | | |
| Omitting Ashburner et al. 2018 | 1376/1315 | 5 | 1.15 | [0.80, 1.66] | 0.76 | 0.45 | 4 | 0.009 | 70% |
| **Omitting** **Bajorek et al. 2016** | 2142/2492 | 5 | 1.30 | [1.01, 1.69] | 2.02 | 0.004 | 4 | 0.48 | 0% |
| Omitting Chaturvedi et al. 2019 | 2276/2598 | 5 | 1.25 | [0.79, 1.98] | 0.96 | 0.34 | 4 | 0.003 | 75% |
| Omitting Piazza et al. 2019 (AF-ALERT) | 2100/2469 | 5 | 1.10 | [0.80, 1.52] | 0.60 | 0.55 | 4 | 0.03 | 63% |
| Omitting Piazza et al. 2023 (AF-ALERT2) | 1953/2276 | 5 | 1.22 | [0.84, 1.77] | 1.03 | 0.30 | 4 | 0.003 | 75% |
| **Omitting** **Silbernagel et al. 2016** | 1893/2245 | 5 | 1.04 | [0.79, 1.35] | 0.27 | 0.79 | 4 | 0.18 | 36% |

**Table S5: Sensitivity analysis**

MD: mean difference; CI: confidence interval; df: degrees of freedom.
